# The Therapeutics of Functional Headache

**Published:** 1875-02-15

**Authors:** Allan McLane Hamilton


### THE THERAPEUTICS OF FUNCTIONAL HEADACHE.

By ALLAN MCLANE HAMILTON, M.D.

*From the Philadelphia Medical Times, Feb. 6th,* 1875.

ALL remedies for the alleviation of the several varieties of this condition may be included under the following heads:

- I. INTERNAL.

  - Cerebral anæmiants.

  - " stimulants.

  - Those diminishing reflex irritations.

  - " " remote local congestion.

  - Cardiac sedatives.

  - Alteratives.

    - Malarial.

    - Syphilitic.

    - Gouty.

    - Rheumatic.

- II. LOCAL.

  - Peripheral irritants.

  - " anæsthesiants.

  - " revulsives.

- III. GALVANISM.

The headaches dependent upon anæmia of the brain result usually from nervous exhaustion. These are the headaches of brain-workers, or may also follow unusual physical fatigue. The distressing persistency of the headache of nervous prostration is the characteristic feature. It is the most protean of all forms, as it may be a close counterfeit of neuralgia, or, on the other hand, may be dull and sub-acute. These patients are usually anæmic and much reduced.

The headaches are associated with vertigo, and oftentimes nausea. There is usually vomiting, and sometimes syncope. The mental powers are exhausted, and the patient who suffers in this way usually awakes unrefreshed by sleep, with dull, heavy pains and a sense of fatigue. There is little disposition to use the mind; the pulse is small and feeble, and there is sometimes a tendency to passive cerebral congestion. The skin is cool during the attack, and the surface may even be moist.

Many of my patients who suffer in this way are women, and the headache is the most distressing when the patients awaken. The use of a cup of tea or coffee is excellent at this time, and I have lately found that cocoa given in the form of a fluid extract is of very great benefit. Messrs. Hazard and Caswell have made for me a fluid extract which is quite concentrated. A drachm or two of this is the dose. The following prescription is a favorite of mine for these headaches:

℞ Strichniæ sulph., gr. i;

Acidi phosph. dil.,

Tr. ferri chlor., aa ʒ vi;

Aquæ camphoræ, ad ℥ iv;—M.

Sig.—A teaspoonful after eating.

The use of diffusible stimulants is in order. We may give the patient the aromatic spirits of ammonia and sherry wine several times a day with good results. Muriate of ammonia is an invaluable remedy in these headaches, particularly in hemicrania; it should be given in very large doses—from ten grains to thirty—every hour until relief is obtained.

A form of headache, spoken of as *hyperæsthetic* by Hanfield Jones, demands opposite treatment, for the administration of stimulants aggravates it greatly. These are the cases where there are redness of the face, tense carotids, injected conjunctivæ, and heat of skin, the patient is very restless, and the mental faculties are confused. These patients have cold hands and feet during the paroxysms, as a rule. There is imperfect nervous stimulation of the heart, and the cerebral vaso-motors are subject to paresis. These patients find it difficult to sleep; there is tossing at night, and the mind is possessed by a myriad of thoughts that chase each other through the brain. The first order of remedies in my table are of value here, and the bromides are the best of them. We may give this prescription and hope for good results, sometimes very immediate ones:

℞ Sodii bromidi, ℥ i;

Fld. ext. ergotæ, ℥ iss;

Aquæ camphoræ, ad ℥ iv;—M.

Sig.—A teaspoonful every three hours, or two teaspoonfuls at night.

I believe the sodic salt to be the most efficacious of all, and the most reliable. Bromide of calcium is next in order, I am convinced, after having given it an extended trial.

In these headaches, cardiac sedatives are of incalculable benefit. Tincture of aconite and veratrum viride will often produce happy results. The continued use of digitalis, combined with zinc, the latter in the form of the oxide, does much to change the character of the circulation.

For the headaches of inebriety I have used since the year 1871 the monobromate of camphor. The results of my experiments I published in the *New York Medical Journal* of August of that year. I am sorry to see that this excellent remedy has fallen into disuse, for it seems to possess hypnotic properties peculiar to itself.

Bourneville, of Paris, has recently called attention to its physiological effects, and I trust its use will be more extended, it having received favor at the hands of this distinguished gentleman. Local depletion, and in some cases general depletion, are necessary. Leeches and cupping relieve the gorged sinuses at the base of the skull. A very common class of headaches are those dependent upon reflex causes. They may be called the *inhibitory* headaches. They go hand in hand with disturbance of digestion, irregularities in the uterine functions, and with other conditions dependent upon eccentric irritations transmitted to the central nervous axis. These headaches partake of all varieties; we may have the well-known sick headache, the headache of dysmenorrhœa, or that associated with an irritable uterus. Of course, our diagnosis will point out the cause very quickly; but oftentimes there are points of irritation we may overlook. Hæmorrhoids may often produce headache, associated with great restlessness and fatigue. Its seat is usually in the frontal region, and it comes on very suddenly.

The uterus will account for two-thirds of the headaches among women, and one of my patients suffers with a common form all have met with undoubtedly. Her uterus is retroverted: mechanical pressure is made upon the rectum to such a degree that the walls of that gut are in contact nearly all the time. A headache is the result, which is persistent and very prostrating. She suffers constantly from constipation, and before I saw her was often in such extremities from retained fæces that she would pass an ivory paper cutter up the rectum, and press the uterus forward. After working in this manner for some time, and using a syringe, she would have an unsatisfactory, ribbon-like, and greatly attenuated stool, and the headache would disappear for several days. These cases are more familiar to the gynæcologist than to the neurologist.

Just as the stomach, when irritated by undigested food, transmits to the brain in gastric epilepsy a morbid irritation, and a convulsion is the consequence, so does it send irritations that are followed by headaches.

We are to meet these conditions therapeutically by special interference and proper remedies.

There is a somewhat rare variety of headache, but an excessively painful one,—I allude to rheumatic headache. The pain is superficial; there is a diffused hyperæsthesia over the scalp, which is very sensitive to touch. The disease may be deeper, and the dura mater be the seat of rheumatic inflammation. This is the rare variety. The external hyperæsthesia is due generally to cold. I have found it amenable in a very few minutes to the faradic current applied by the wire brush. Of course, alterative medicines will be required should there be much constitutional participation.

Headaches are associated with syphilis in nearly every instance. Oftentimes there are deep organic changes, sometimes of the dura mater, or there may exist a tumor. The headache is intense, localized, and not always attended by acceleration of pulse. It is needless to say it is worse at night. Inunctions of mercurial ointment have met my anticipations in many cases. In old cases we naturally resort to specific medicine.

The suboccipital headache of malaria is often uncontrolled by quinine alone. The combination of arsenious acid is of great use, and the addition of a small quantity of belladonna increases still more its effect.

Neuralgia is dependent upon so many causes that it will be difficult to consider its therapeutical indications without going very deeply into the history and etiology of the disease. The peripheral forms, however, deserve notice in a paper devoted to the discussion of functional diseases, and, as these are very commonly met with, particularly the facial form, it might be apropos to speak of a few serviceable remedies. I know of none so good as iron, quinine, and belladonna, or arsenic in some one of its forms. This prescription is a good one, I think, for it contains three of these agents:

℞ Morph. sulph., gr. vi;
Ext. belladonnæ,
Ext. nucis vomicæ, aa gr. xii;
Ferri et quiniæ citrat., ʒ iiss.—M.
Ft. massa et divid. in pil. No. xlviii, one t. i. d.

Strychnia is of great benefit in the anæmic variety of this disorder.

Peripheral neuralgia is treated most successfully by local applications, and among these come galvanism, chloroform, irritant applications, such as blisters, etc., and the actual cautery. The application of chloroform and of bisulphide of carbon has been recommended by several English writers. One of these substances should be poured upon a piece of cotton, and the same placed in a wide-mouthed bottle. The mouth of the bottle is to be then held against the most painful part of the face for a few minutes. A few drops of nitrite of amyl inhaled have often stopped a severe neuralgia.

The hypodermic syringe is so much used that it would be unnecessary to allude to it. I would only speak of certain solutions that have been tried with different degrees of success.

Morphine stands prominently forward as the best. Combined with atropine it is perhaps more efficacious than when injected alone. In neuralgia, chloroform injected hypodermically has been highly recommended by Roberts Bartholow. I think its greatest fault is the production of abscesses. I have used it several times, but have always had unpleasant consequences of this kind. The irritant nature of this drug forbids its application to the skin even locally. We have all seen the blistering produced by the local application. How much more intense must be its action beneath the skin!

Blistering the skin and afterwards applying morphine to the denuded surface, is effectual in stopping some forms of peripheral neuralgia.

I have lately tried, with the most satisfactory results, the local application of the ether-spray by the atomizer. Freezing of the skin just anteriorly to the ear will cut short a violent attack of facial neuralgia in a few moments.

In certain forms of facial neuralgia, particularly where there are points of irritation, the actual cautery-iron, brushed over these points, will cure the patient.

Perhaps one of our best remedies is electricity. In the form of galvanism we may affect the cervical sympathetic, diminish the cerebral hyperæmia, or by stronger currents increase it. The poles should be held over the nuchæ or lower down, and over the mastoid bone, or upon both temples. In neuralgia the positive pole may be held just back of the ear, and the negative passed over the several branches of the fifth nerve.

The faradic current often relieves many headaches, particularly if they are diffused over the scalp, and if they are aggravated by heat to the head, or by pressure.

The application of cold is one of the best local means we have to modify or stop headache, particularly if it be of the hyperæsthetic variety. Bladders filled with ice, cold douches, and other expedients enable us to successfully combat it.

The organic headaches deserve mention by themselves, so I will not venture upon such a wide field. In all cases of this kind it is a symptom, and while attempting to relieve it, we must not forget that there is usually a cause.

